# Chylothorax as the Initial Manifestation of Small Cell Lung Carcinoma

**DOI:** 10.1002/rcr2.70202

**Published:** 2025-05-05

**Authors:** Sathish Krishnan, Anam Naumaan, Venu Pararath Gopalakrishnan

**Affiliations:** ^1^ Division of Pulmonary and Critical Care Medicine Community Health Network Indianapolis Indiana USA; ^2^ Division of Anatomic and Clinical Pathology Community Health Network Indianapolis Indiana USA; ^3^ University of Massachusetts Chan Medical School Worcester Massachusetts USA

**Keywords:** chylothorax, chylothorax in lung cancer, chylothorax in malignancy, non‐traumatic chylothorax, pleural catheter in chylothorax

## Abstract

Chylothorax, the accumulation of lymphatic fluid in the pleural space, is a rare condition often linked to trauma, surgery or malignancy. We present the case of a 63‐year‐old female with a significant smoking history who was diagnosed with chylothorax as the initial manifestation of small cell lung carcinoma (SCLC). Diagnostic workup, including pleural fluid analysis and imaging, confirmed the diagnosis and revealed mediastinal lymphadenopathy and bronchial narrowing. The patient underwent chemotherapy, immunotherapy and management of recurrent effusions but ultimately succumbed to pneumonia and septic shock. Recognising chylothorax as a potential initial manifestation and understanding the risks of prolonged chyle drainage are crucial for effectively managing such complex cases.

## Graphical abstract

Chylothorax can indeed be an initial manifestation of lung cancer. Non‐traumatic and non‐iatrogenic chylothorax requires thorough investigation, with computed tomography (CT) of the chest being a crucial diagnostic tool. Prolonged drainage of chylothorax can lead to exaggerated immunosuppression in malignancy.

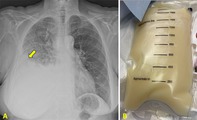

## 1 Introduction

Chylothorax is characterised by the accumulation of chyle in the pleural space, which arises from disruption or obstruction of the thoracic duct. It is most commonly associated with trauma, surgical complications or malignancies such as lymphoma, while its occurrence in small cell lung carcinoma (SCLC) is exceedingly uncommon [1, 2]. In the context of malignancy, chylothorax results from direct invasion or compression of the thoracic duct by tumour masses or metastatic lymph nodes [3]. This condition presents unique diagnostic and therapeutic challenges, requiring a multidisciplinary approach for effective management.

## 2 Case Report

**FIGURE 1:**
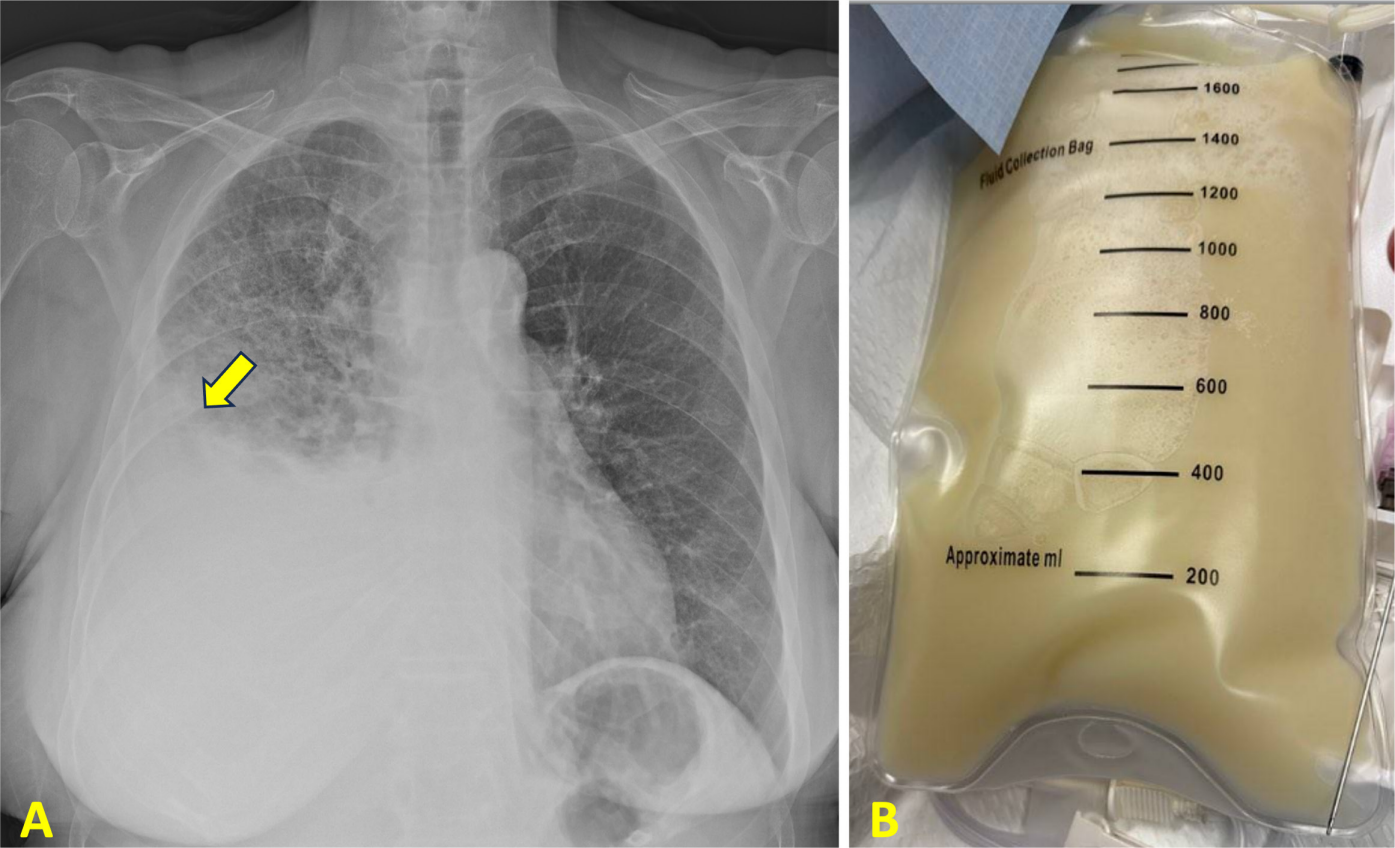
(A) Chest X‐ray showing large right pleural effusion with meniscus sign (yellow arrow). (B) Thoracentesis revealing chylous fluid with a milky‐white appearance.

A 63‐year‐old female with a 45‐pack‐year smoking history presented to the emergency department (ED) with a two‐week history of progressive shortness of breath and cough. She did not report recent trauma or surgical interventions. On examination, she was tachypneic but not hypoxemic. Physical findings included decreased breath sounds on the right side. A chest X‐ray revealed a right‐sided pleural effusion confirmed by thoracentesis, which yielded 1.5 L of milky fluid (Figure 1). Pleural fluid analysis indicated a chylothorax with a triglyceride level of 434 mg/dL and a cholesterol level of 154 mg/dL (Table 1).

**TABLE 1:** Pleural fluid characteristics.

Variables	Values	Reference
Total white cells	1068/μL	< 1000/μL
Lymphocytes	82%	Not established
pH	7.48	7.4–7.6
Lactate Dehydogenase (LDH)	83 U/L	120–246 U/L
Protein	2.4 g/dL	6.3–8.2 g/dL
Gram stain and culture	Negative	Negative
Triglycerides	434 mg/dL	< 110 mg/dL
Cholesterol	154 mg/dL	< 200 mg/dL
Cytology	Rare mesothelial cells with lymphocytes. No malignant cells.	Normal cells with no atypical features

**FIGURE 2:**
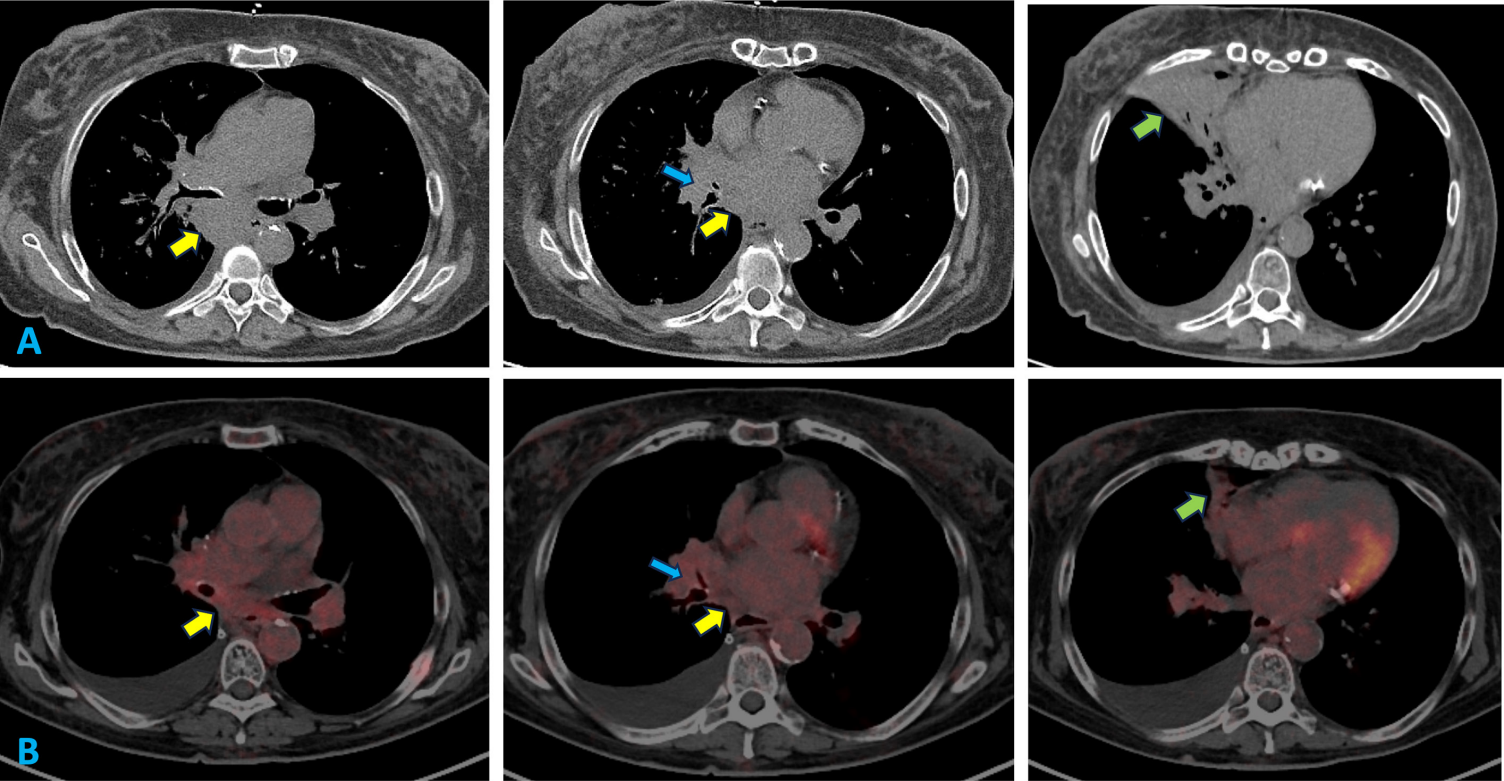
Upper panel (A) Computed tomography images showing enlarged subcarinal (yellow arrows) and right hilar (blue arrow) nodes, causing narrowing of the right middle lobe bronchus and collapse of the right middle lobe (green arrow). Lower panel (B) Positron Emission Tomography images showing a decrease in size of subcarinal (yellow arrows) and right hilar (blue arrow) nodes, and improvement in the collapse of the right middle lobe (green arrow).

The patient initially improved but returned to the ED days later with worsening dyspnea. Imaging revealed recurrent right pleural effusion. Repeat thoracentesis produced 1.2 L of fluid with similar characteristics. Given her smoking history, a computed tomography (CT) scan was performed, showing bilateral hilar and mediastinal adenopathy, with severe narrowing of the right middle lobe bronchus, and partial atelectasis and infiltrates of the right middle lobe (Figure 2A). Bronchoscopy with endobronchial ultrasound‐guided biopsy confirmed extensive‐stage small cell carcinoma. The patient was started on carboplatin and etoposide, later supplemented with atezolizumab.

She experienced rapid reaccumulation of pleural fluid causing dyspnea, and therapeutic thoracentesis was inadequate, necessitating the placement of an indwelling pleural catheter, which drained 700–800 mL daily. Owing to the rapid build‐up of fluid and high‐volume output, thoracoscopy with pleurodesis was not considered suitable. She also chose not to pursue dietary modifications. The patient was therefore advised to continue daily drainage via the catheter to alleviate dyspnea. In addition, she elected against dietary modifications. During follow‐up in the pulmonary clinic, the patient reported a reduction in fluid drainage output. As a result, she was initially advised to decrease the frequency of drainage to three times per week. With continued improvement, the recommendation was further adjusted to twice‐weekly drainage. Six weeks later, she reported an output of about 550 mL twice a week.

Follow‐up positron emission tomography that was obtained 6 weeks later showed partial response to therapy, with reduced lymphadenopathy and improved bronchial narrowing (Figure 2B). However, she presented 2 weeks later with fever, chills and worsening respiratory symptoms. Work‐up showed neutrophilic leukocytosis (white cell count: 28.8 K/cumm with 87% neutrophils) and thrombocytosis (1020 K/cumm). Imaging revealed bilateral lower lobe infiltrates suggestive of pneumonia. Blood cultures identified *Pseudomonas aeruginosa* . Despite aggressive management, she rapidly deteriorated due to septic shock, ultimately leading to cardiac arrest and death.

## 3 Discussion

Chylothorax is characterised by leakage of chyle into the pleural space and it accounts for 2% of all pleural effusions [1]. Chylothorax is typically classified into traumatic and non‐traumatic etiologies. Traumatic causes include penetrating or blunt chest trauma, as well as surgical interventions involving the thoracic cavity. Non‐traumatic chylothorax is most commonly caused by malignancy, with lymphoma being the leading contributor. Non‐malignant causes include yellow nail syndrome, sarcoidosis, histoplasmosis, tuberculosis and Castleman disease. In patients with lung cancer, it occurs usually as a complication of thoracic surgery or radiotherapy. Chylothorax as an initial manifestation of small cell lung cancer (SCLC) is exceedingly rare [2].

In malignancies like SCLC, it can occur due to direct invasion or compression of the thoracic duct by tumour masses or metastatic lymph nodes. The high propensity for SCLC to metastasize to mediastinal lymph nodes increases the risk of thoracic duct involvement, thereby precipitating chylothorax [3]. The thoracic duct originates from the cisterna chyli, the coalescence of intra‐abdominal and lower extremities lymphatic channels anterior to the second lumbar vertebra. It carries triglycerides including chylomicrons, T‐lymphocytes, immunoglobulins and fat‐soluble vitamins in the form of chyle. As it ascends into the mediastinum, the thoracic duct crosses from right to left of midline at the level of the fourth to sixth thoracic vertebrae [4]. Compression of the thoracic duct at this level is considered the mechanism of development of chylothorax in conditions like lymphoma, sarcoidosis, histoplasmosis, tuberculosis and Castleman disease [5]. In our patient, we suspect that the enlarged subcarinal node was causing compression of the thoracic duct at this level.

The diagnosis of chylothorax is confirmed through pleural fluid analysis, typically revealing a milky appearance with elevated triglyceride levels (> 110 mg/dL), a cholesterol level of < 200 mg/dL, and a predominance of lymphocytes [6]. Unexplained chylothorax, particularly in the absence of recent trauma or thoracic surgery should prompt concern for malignancy. A thorough evaluation, primarily with a computed tomography of the chest is recommended. Advanced imaging techniques, including lymphangiography, can further delineate the site of chyle leakage and guide therapeutic interventions [7].

There is a paucity of data on the optimal management of chylothorax associated with malignancy. It involves addressing both the underlying malignancy and the chyle leak. Initial conservative measures include dietary modifications, such as a low‐fat diet enriched with medium‐chain triglycerides or total parenteral nutrition, to reduce chyle production [8]. Pharmacological interventions, like octreotide, have been employed to decrease lymphatic flow, though evidence supporting their efficacy in malignancy‐associated chylothorax is limited [9].

Recurrent and rapid reaccumulation of chylothorax may necessitate the placement of an indwelling pleural catheter. However, prolonged chest tube drainage can lead to nutritional deficiencies and immunosuppression, as chyle is rich in T‐lymphocytes, immunoglobulins, electrolytes, proteins and chylomicrons [3]. This presents unique challenges in patients with malignancy, who may already be immunocompromised due to both the disease and chemotherapy. Notably, the 90‐day mortality rate associated with this condition has been reported to be as high as 82% [4]. In our patient, prolonged high‐volume drainage may have exacerbated immunosuppression, potentially contributing to pseudomonas bacteremia and sepsis.

In cases where conservative management fails, invasive procedures may be warranted. Thoracic duct ligation or embolization are surgical options aimed at sealing the site of leakage [10]. These options need to be considered early, particularly in patients with high‐volume output and additional factors causing malnutrition and immunosuppression. Although surgical interventions are generally effective in managing traumatic chylothorax, their role in malignancy‐associated chylothorax remains uncertain. Given the unclear efficacy of both octreotide and surgical approaches in this context, we opted not to pursue these options for our patient. Instead, the decision was made to focus on treating the underlying malignancy and closely monitor the response. The choice between conservative and surgical interventions should be individualised, considering factors such as the patient's overall condition, the volume of chyle output, and the response to initial therapies [11].

In conclusion, Chylothorax can be an initial manifestation of lung cancer. This case underscores the importance of maintaining a high index of suspicion for timely diagnosis of underlying malignancy. A multidisciplinary approach to management is crucial, focusing on both treating the malignancy and addressing complications of chyle leak. Recognising the immunosuppressive risks of prolonged drainage is vital to optimising patient care and preventing adverse outcomes.

## Author Contributions

**Sathish Krishnan:** conceptualization, supervision, writing – original draft. **Anam Naumaan:** resources, writing – review and editing. **Venu Pararath Gopalakrishnan:** resources, visualization, writing – review and editing.

## Ethics Statement

The authors attest that the submitted manuscript conforms to the ICMJE Recommendations for the Conduct, Reporting, Editing, and Publication of Scholarly Work in Medical Journals. Institutional Review Board approval not applicable given type of manuscript. The authors declare that written informed consent was obtained from the patient's healthcare power of attorney for patient information and images to be published.

## Conflicts of Interest

The authors declare no conflicts of interest.

## Data Availability

Data sharing not applicable to this article as no datasets were generated or analysed during the current study.
